# Elevation gradient of soil bacterial communities in bamboo plantations

**DOI:** 10.1186/s40529-016-0123-0

**Published:** 2016-02-29

**Authors:** Yu-Te Lin, Chih-Yu Chiu

**Affiliations:** Biodiversity Research Center, Academia Sinica, Nankang, Taipei, 11529 Taiwan

**Keywords:** Bamboo, Soil, 16S rRNA genes, Elevation, Bacterial diversity

## Abstract

**Background:**

Elevation trends of macro organisms have long been well studied. However, whether microbes also exhibit such patterns of elevation change is unknown. Here, we investigated the changes in bamboo forest soil bacterial communities along six elevation gradients, from 600 to 1800 m a.s.l. in Mt. Da-an, a subtropical montane area in Nantou county at central Taiwan.

**Results:**

Data from 16S rRNA gene clone libraries revealed that more than 70 % of the six communities contained *Acidobacteria* and *Proteobacteria*, although the relative abundance differed. Nonmetric multidimensional scaling analysis of the distribution of operational taxonomic units showed differences in bamboo soil bacterial communities across gradients. The bacterial communities at 1000 and 1200 m showed greater diversity than the communities at both lower (600 and 800 m) and higher (1400 and 1800 m) elevations. In contrast to the bacterial community trend, soil C and N and microbial biomass properties increased linearly with elevation.

**Conclusion:**

The bamboo soil bacterial community could interact with multiple factors such as soil organic matter content and temperature, for differences in composition and diversity with change in elevation.

## Background

Understanding the responses of soil communities across elevation gradients has long been a fascinating topic for ecologists. The composition of macro organisms along elevation gradients have been well studied (Herzog et al. 2005; Rahbek 2005; Kreft and Jetz 2007; McCain 2009). However, the distribution of microorganisms might not follow the same ecological rules as for macro organisms. For instance, the number of total bacteria, methanotrophic bacteria and ammonia-oxidizing archaea was negatively correlated with increasing elevation (Ma et al. 2004; Giri et al. 2007; Zhang et al. 2009), but the content of Gram-negative bacteria increases with increasing elevation in the Austrian central Alps (Margesin et al. 2009). Also, bacterial diversity decreased with elevation in the mountains of the southwestern United States (Bryant et al. 2008). However, in eastern Peru, the community diversity did not show a significant elevation gradient (Fierer et al. 2011). In South Korea, higher diversity occurs at high and low elevations, with minimum richness at middle elevations (Singh et al. 2014). Our understanding of major determinants of the distribution of bacteria is still largely limited. Considering the essential roles of microorganisms in many biogeochemical cycles in ecosystems, more studies on the bacterial trends among different mountain ecosystems are needed.

In East Asia, bamboo is one of the most important forest resources and can be used as construction or furniture material. Young bamboo shoots are also in high demand as a healthy food. Regular management, such as removal of understory vegetation as well as tillage and fertilizer application, is often used to maintain and increase bamboo production.

In the present study, we collected soil samples from moso bamboo plantations along six elevations, from 600 to 1800 m a.s.l., in central Taiwan. This transect provides an opportunity to study elevation distribution without the effects of different vegetation. Previously, we found the high-elevation bamboo plantations (1200 and 1400 m) significantly differed from low elevation plantations (600, 800, and 1000 m), with higher soil C and N contents, higher concentrations of soil soluble organic C and N, and a greater amount of soil microbial biomass C and N (Huang et al. 2014). However, the bacterial community trend along elevation gradients is still unknown. Using 16S rRNA gene clone library analysis, we attempted to elucidate the responses of the structure and diversity of the bamboo soil bacterial community to elevation gradients.

## Methods

### Site description and soil sampling

The study site was located at Mt. Da-an, a subtropical montane area in Nantou County, central Taiwan (23°42′N, 120°41′E). Soil samples were collected from moso bamboo (*Phyllostachys edulis*) plantations along a county road with an increasing altitudinal gradient, 600, 800, 1000, 1200, 1400 and 1800 m a.s.l. The six sampling gradients are all dominated by moso bamboo with few understory plants. The bamboo plantations in this area were established simultaneously around the 1960s. Both the culm density and culm age of the selected moso bamboo plantations were similar between elevations. However, aboveground bamboo biomass increased with elevation (Chen et al. 2014). The soils are well drained and characterized as Entisols. The parent material is sandstone and shale. The soils are loam with pH 3.9–4.4. Other properties of soils are reported in Table 1.

**Table 1:** Soil chemical and physical properties of study sites

Elevation (m)	pH	Organic C (g kg^−1^)	Total N (g kg^−1^)	C/N
600	4.0	25.3	2.4	10.5
800	3.9	35.8	3.6	9.9
1000	3.8	39.3	3.8	10.3
1200	3.7	64.9	6.1	10.6
1400	3.4	63.8	5.2	12.3
1800	4.4	151.3	10.2	14.8

At each elevation, three 25 × 25 m plots were established along transect lines in January 2012. The soil samples were collected in winter to avoid differences caused by seasonal changes. Within each plot, three subsamples were collected by use of a soil auger 8 cm in diameter and 10 cm deep and were combined. Visible detritus materials, such as roots and litter, were manually removed prior to passing soil through a 2-mm sieve. Soils were then stored at −20 °C, and extraction of soil community DNA was performed within 2 weeks.

## 16S rRNA gene clone library construction and sequencing

The 16S rRNA gene clone libraries were constructed as described (Lin et al. 2010). In brief, soil community DNA was extracted by using the PowerSoil^®^ Soil DNA Isolation kit (MoBio Industries, Carlsbad, CA, USA) following the manufacturer’s instructions. The bacterial 16S rRNA genes were amplified by PCR with the primer set 27F and 1492R (Lane 1991). After 15 cycles, the PCR products were cloned by using the TOPO TA cloning kit (Invitrogen, Carlsbad, CA, USA) and the pCR2.1 vector. White colonies on selective Luria–Bertani (LB) agar plates were picked into 96-well blocks containing 1 ml LB broth plus kanamycin (50 µg ml^−1^) and grown overnight. Sterile glycerol was added to a final concentration of 10 %, and an aliquot was transferred to a 96-well sequencing block. Both the sequencing and the original culture blocks were stored at −80 °C.

### DNA sequencing and sequence analyses

Bacterial clones were partially sequenced with the primer 27F. Sequence analysis involved an ABI PRISM Big Dye Terminator cycle sequencing ready reaction kit (Applied Biosystems, Foster City, CA, USA) and an ABI 3730 Genetic Analyzer (Applied Biosystems) following the manufacturer’s instructions. Sequences were analyzed with the Mallard and Pintail programs to test for chimeras (Ashelford et al. 2005, 2006). The entire clone sequences obtained in the study have been deposited to GenBank (accession numbers KJ407398-KJ408214 and KM108145-KM108301).

Taxonomic assignment of sequences from the clone library was made using the naïve Bayesian rRNA classifier (Wang et al. 2007) in the Ribosomal Database Project (RDP) (http://rdp.cme.msu.edu/index.jsp). Diversity estimates, including Shannon diversity index, Chao1 estimator and rarefaction analysis, were calculated for operational taxonomic units (OTUs) with 97 % 16S rRNA gene sequence similarity by using the program DOTUR (Schloss and Handelsman 2005). PRIMER V6 (PRIMER-E, Lutton, Ivybridge, UK) was used for non-metric multi-dimensional scaling (NMDS) generated with Bray-Curtis similarity of sequence data. Mantel tests as implemented in PRIMER v6 were also assessed to analyze the relationships between phylogenetic distances of bacterial communities and soil properties.

## Results

### Phylogenetic groups of bacterial community

**Fig. 1:**
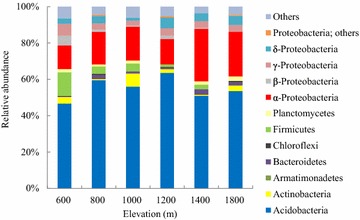
Relative abundance of phylogenetic groups in 16S rRNA gene libraries along elevational gradients

About 50–60 clones of 16S rRNA genes were derived from each of the three replicate samples. The sequences from replicates of each elevation were then combined for further analysis. A total of 151–182 bacterial sequences were obtained from each of six elevations. Phylogenetic analysis revealed that the communities were composed of 11 bacterial groups. The *Acidobacteria* (comprising 46–64 % of all clones from each elevation) and *Proteobacteria*-affiliated clones (24–37 %) were the two most abundant phyla (Fig. 1). The relative abundance of *Actinobacteria* and *Firmicutes* in the community at 1000 and 600 m, respectively, was higher than that in other communities. Other phyla including *Bacteroidetes*, *Chloroflexi*, *Cyanobacteria*, *Gemmatimonadetes*, *Planctomycetes* and *Verrucomicrobia*, were all less abundant and accounted for less than 5 % of the clones (Fig. 1).

Within the *Acidobacteria*, GP1, 2 and 3 were all abundant groups in the six communities, but the relative abundance of GP2 at 1400 m, and GP3 at 600, 800 and 1800 m was less than 10 % (Table 2). The relative abundance of *Bacillales* was higher at 600 m than that in other communities (Table 2). Within the α-*Proteobacteria*, *Rhizobiales* was more abundant in the communities at 1400 and 1800 m. The relative abundance of *Rhodospirillales* was higher at 1000 m than that in the other communities (Table 2).

**Table 2:** The 10 most abundant phylogenetic groups detected in bamboo soil communities along elevation gradients

Phylogenetic group	Percentage of clones					
	600 m	800 m	1000 m	1200 m	1400 m	1800 m
*Acidobacteria*						
GP1^a^	25.3	37.0	19.8	29.8	17.9	18.9
GP2	10.2	8.7	17.6	17.2	9.0	23.3
GP3	6.6	7.5	15.9	12.6	21.4	3.8
GP5	3.6	2.9	1.1	2.0	2.1	1.9
*Bacteroidetes*						
*Sphingobacteriales*	0.6	2.3	1.1	0.7	3.4	1.9
*Firmicutes*						
*Bacillales*	10.8	3.5	0.5	1.3	0.7	0.0
Planctomycetes						
*Planctomycetales*	3.0	0.6	1.6	0.0	3.4	2.5
α-*Proteobacteria*						
*Rhizobiales*	5.4	12.1	9.3	10.6	20.0	20.1
*Rhodospirillales*	2.4	4.6	8.2	3.3	4.8	3.1
γ-*Proteobacteria*						
*Xanthomonadales*	4.8	2.3	2.2	1.3	3.4	3.8

^a^GP, group

### Bacterial diversity

**Fig. 2:**
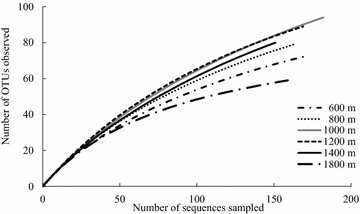
Rarefaction curve analysis for the bamboo soil libraries with operational taxonomic units (OTUs) with 97 % nucleotide sequence similarity

The community diversity indices were calculated based on the OTUs with 97 % sequence similarity. Although we observed no clear trends in diversity indices (Table 3), analyses of rarefaction curves suggested that communities at 1000 and 1200 m were the most diverse, and the communities at the lowest elevation of 600 m and the highest elevation of 1800 m were the least diverse (Fig. 2).

**Table 3:** Diversity of soil bacterial communities detected in 16S rRNA gene clone libraries^a^

Measure of diversity	Elevation (m)					
	600	800	1000	1200	1400	1800
No. of sequences	169	173	182	151	163	159
No. of OTUs	89	73	94	80	79	59
Shannon^b^	4.29	3.93	4.27	4.07	4.08	3.82
Chao 1	132	112	151	134	124	80
95 % Chao 1	110–179	90–162	123–209	106–192	100–176	67–117

^a^Calculations were based on operational taxonomic units (OTUs) with 97 % 16S rRNA gene sequence similarity

^b^Shannon diversity index (*H*)

### Bacterial community comparison

**Fig. 3:**
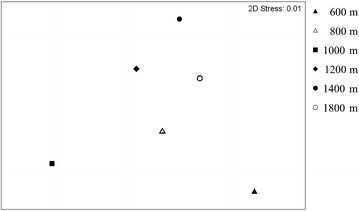
Non-metric multi-dimensional scaling plot of six plantation soil bacterial communities based on distribution of OTUs. The OTUs were at an evolutionary distance of 0.03

On NMDS analysis of the library, the composition of bamboo soil bacterial community differed across elevation gradients (Fig. 3). The communities in 600 and 1000-m soils were more separated from other communities. Examining the distribution of OTUs also revealed differences in composition. The distribution of the 10 most abundant OTUs, affiliated with *Acidobacteria* and α-*Proteobacteria*, differed by elevation (Table 4). OTU 8 could be found in all six communities, with OTU 29 not found in soils at 600 and 1800 m. Likewise, OTUs 23 and 50 were present only in soils lower than 1000 m. OTU distribution was not significantly correlated associated with environmental parameters, including elevation, organic C, total N, C/N ratio and soil pH (*P* > 0.05; data not shown).

**Table 4:** Relative abundance and phylogenetic affiliation of the ten most abundant OTUs^a^

OTU	No. of sequences	Affiliation	Elevation (m)					
			600	800	1000	1200	1400	1800
8	50	*Rhodoplanes*	8	7	10	8	8	9
95	28	*Acidobacteria*	0	7	9	9	3	0
1	25	*Acidobacteria*	6	7	2	3	5	2
49	20	*Bradyrhizobium*	1	7	1	1	10	0
53	15	*Acidobacteria*	3	4	3	5	0	0
10	14	*Acidobacteria*	2	0	1	1	5	5
35	14	*Acidobacteria*	5	4	1	2	1	1
23	11	*Rhodospirillaceae*	1	7	3	0	0	0
50	11	*Acidobacteria*	4	6	1	0	0	0
142	11	*Acidobacteria*	0	1	2	2	2	4

^a^OTUs with 97 % 16S rRNA gene sequence similarity

## Discussion

In the present study, along six elevation gradients from 600 to 1800 m a.s.l. in bamboo forests, the soil bacterial communities at 1000 and 1200 m a.s.l. showed higher levels of diversity than those at both lower and higher elevations. The diversity differed from the monotonic and unimodal patterns observed for macro organisms (Rahbek 2005; McCain 2009). This result is similar to the changes in bacterial diversity with elevation on Mount Fuji (Singh et al. 2012), suggesting that the pattern of bamboo soil bacterial diversity along elevation gradients is different from that of plants and animals. With phospholipid fatty acid and denaturing gradient gel electrophoresis analysis, the microbial community the low and high elevations (Chang et al. 2016). In a comprehensive analysis of soil bacterial communities across six elevations representing six vegetation types from forest to alpine tundra in Mt. Changbai, China, pH was the best predictor of community composition, and vegetation could have indirectly affected the communities by altering soil C and N status across elevation gradients (Shen et al. 2013). In the present study, the range of soil pH was narrow (3.4-4.4), and pH was not significantly correlated with bacterial community structure. Because small pH changes could cause differences in bacterial communities within short and long distances (Yergeau et al. 2010; Sagova-Mareckova et al. 2011), the effects of pH on the bamboo soil community in this region still need to be further investigated.

Disturbance can be a major factor affecting the bamboo soil bacterial diversity. In recent years, the major production area for timber and bamboo shoots has changed from 600 and 800 m to 1000 and 1200 m in this region. However, the plantations we studied possessed comparable culm density and ages, which indicates similar bamboo productivity and levels of disturbance. Because of the different owners, the bamboo plantations might have been subjected to different fertilizer application and stem harvest, and determining the exact influence of human activity is difficult. One study showed that the effects of disturbance could affect microbial communities across time (Keiser et al. 2011), suggesting the need for further elucidation in these communities.

The *Acidobacteria* were the most abundant group. They also predominate in other ecosystems, including agricultural systems and subtropical and tropical forests (Jangid et al. 2011; Araujo et al. 2012; Meng et al. 2013). *Acidobacteria* are generally considered as oligotrophs (Nemergut et al. 2010) and versatile heterotrophs, exhibiting slow metabolic rates under low-nutrient conditions (Ward et al. 2009). Bamboo could release a large number of allelopathic compounds. The heterotrophic ability to survive with allelopathic compounds could be further explored.

*Proteobacteria* were also abundant in these communities, with α-*Proteobacteria* the most abundant across different elevations. Within α-*Proteobacteria*, many sequences were related to the *Rhizobiales* and *Rhodospirillales*, indicating the potential role for N_2_ fixation, organic matter decomposition and plant growth promotion in the soils (Zhang and Xu 2008; Yarwood et al. 2009). Higher soil C/N ratio in higher elevations may indicate lower amounts of available nitrogen and requirement for N_2_-fixing bacteria in the community. The relatively high abundance of *Rhizobiales*-affiliated OTUs at 1400 and 1800 m was associated with high soil C/N ratio, which is an important factor to predict community structure (Chu et al. 2010).

The relative abundance of *Bacteroidetes* was higher at 1400 m than that at other elevations. Known as copiotrophic bacteria, *Bacteroidetes* species tend to be found in nutrient-rich environments (Fierer et al. 2007). High elevation with high amounts of organic C and lower decomposition rates could provide a favorable condition for the growth of *Bacteroidetes*. Our previous study also revealed a higher abundance of *Bacteroidetes* in a cedar forest than adjacent bamboo forest soils (Lin et al. 2013). These results suggest that the *Bacteroidetes*, as well as the phyla *Acidobacteria* and *Proteobacteria*, could play an important role in the soil bacterial community.

## Conclusion

In conclusion, our study revealed that bacterial diversity of bamboo soil communities were lower at both lower and higher elevations in this mountain area of central Taiwan, with greater diversity at 1000 and 1200 m. As well, the community structure differed by elevation. Considering the low sequence number in the clone library, further studies with more sequences and using next generation sequencing technique (e.g., pyrosequencing) are needed to address the responses of the bamboo soil community along elevation gradients.

## References

[CR1] Araujo J, de Castro A, Costa MMC, Togawa RC, Pappas GJ, Quirino BF, Bustamante MMC, Williamson L, Handelsman J, Krüger RH (2012). Characterization of soil bacterial assemblies in Brazilian savanna-like vegetation reveals *Acidobacteria* dominance. Microb Ecol.

[CR2] Ashelford KE, Chuzhanova NA, Fry JC, Jones AJ, Weightman AJ (2005). At least 1 in 20 16S rRNA sequence records currently held in public repositories is estimated to contain substantial anomalies. Appl Environ Microbiol.

[CR3] Ashelford KE, Chuzhanova NA, Fry JC, Jones AJ, Weightman AJ (2006). New screening software shows that most recent large 16S rRNA gene clone libraries contain chimeras. Appl Environ Microbiol.

[CR4] Bryant JA, Lamanna C, Morlon H, Kerkhoff AJ, Enquist BJ, Green JL (2008). Microbes on mountainsides: contrasting elevational patterns of bacterial and plant diversity. Proc Natl Acad Sci USA.

[CR01] Chang EH, Chen TH, Tian G, Chiu CY (2016) The effect of altitudinal gradient on soil microbial community activity and structure in moso bamboo plantations. Appl Soil Ecol 98:213–220

[CR5] Chen TH, Chiu CY, Xie ZY, Wang S (2014). Growth characteristics of moso bamboo (*Phyllostachys edulis*) plantations at various altitudes in Da-an area, Nantou County. Quart J Chin For.

[CR6] Chu H, Fierer N, Lauber CL, Caporaso JG, Knight R, Grogan P (2010). Soil bacterial diversity in the Arctic is not fundamentally different from that found in other biomes. Environ Microbiol.

[CR8] Fierer N, Bradford MA, Jackson RB (2007). Toward an ecological classification of soil bacteria. Ecology.

[CR9] Fierer N, McCain CM, Meir P, Zimmermann M, Rapp JM, Silman MR, Knight R (2011). Microbes do not follow the elevational diversity patterns of plants and animals. Ecology.

[CR10] Giri DD, Shukla PN, Kashyap S, Singh P, Kashyap AK, Pandey KD (2007). Variation in methanotrophic bacterial population along an altitude gradient at two slopes in tropical dry deciduous forest. Soil Biol Biochem.

[CR11] Herzog SK, Kessler M, Bach K (2005). The elevational gradient in Andean bird species richness at the local scale: a foothill peak and a high-elevation plateau. Ecography.

[CR12] Huang CY, Jien SH, Chen TH, Tian G, Chiu CY (2014). Soluble organic C and N and its relationships with soil organic C and N and microbial characteristics in moso bamboo (*Phyllostachys edulis*) plantations along an elevation gradient in central Taiwan. J Soils Sediments.

[CR13] Jangid K, Williams MA, Franzluebbers AJ, Schmidt TM, Coleman DC, Whitman WB (2011). Land-use history has a stronger impact on soil microbial community composition than aboveground vegetation and soil properties. Soil Biol Biochem.

[CR14] Keiser AD, Strickland MS, Fierer N, Bradford MA (2011). The effect of resource history on the functioning of soil microbial communities is maintained across time. Biogeosciences.

[CR15] Kreft H, Jetz W (2007). Global patterns and determinants of vascular plant diversity. Proc Natl Acad Sci USA.

[CR16] Lane DJ, Stackebrandt E, Goodfellow M (1991). 16S/23S rRNA sequencing. Nucleic acid techniques in bacterial systematics.

[CR17] Lin YT, Huang YJ, Tang SL, Whitman WB, Coleman DC, Chiu CY (2010). Bacterial community diversity in undisturbed perhumid montane forest soils in Taiwan. Microb Ecol.

[CR18] Lin YT, Tang SL, Pai CW, Whitman WB, Coleman DC, Chiu CY (2013). Changes in the soil bacterial communities in a cedar plantation invaded by moso bamboo. Microb Ecol.

[CR19] Ma X, Chen T, Zhang G, Wang R (2004). Microbial community structure along an altitude gradient in three different localities. Folia Microbiol.

[CR20] Margesin R, Jud M, Tscherko D, Schinner F (2009). Microbial communities and activities in alpine and subalpine soils. FEMS Microbiol Ecol.

[CR21] McCain CM (2009). Vertebrate range sizes indicate that mountains may be ‘higher’ in the tropics. Ecol Lett.

[CR22] Meng H, Li K, Nie M, Wan JR, Quan ZX, Fang CM, Chen JK, Gu JD, Li B (2013). Responses of bacterial and fungal communities to an elevation gradient in a subtropical montane forest of China. Appl Microbiol Biotechnol.

[CR23] Nemergut DR, Cleveland CC, Wieder WR, Washenberger CL, Townsend AR (2010). Plot-scale manipulations of organic matter inputs to soils correlate with shifts in microbial community composition in a lowland tropical rain forest. Soil Biol Biochem.

[CR24] Rahbek C (2005). The role of spatial scale and the perception of large-scale species-richness patterns. Ecol Lett.

[CR02] Sagova-Mareckova M, Omelka, M. Cermak L, Kamenik Z, Olsovska J, Hackl, E, Kopecky J, Hadacek, F (2011) Microbial communities show parallels at sites with distinct litter and soil characteristics. Appl Environ Microbiol 77:7560–7567 10.1128/AEM.00527-11 PMC3209186 21926225

[CR25] Schloss PD, Handelsman J (2005). Introducing DOTUR, a computer program for defining operational taxonomic units and estimating species richness. Appl Environ Microbiol.

[CR26] Shen C, Xiong J, Zhang H, Feng Y, Lin X, Li X, Liang W, Chu H (2013). Soil pH drives the spatial distribution of bacterial communities along elevation on Changbai Mountain. Soil Biol Biochem.

[CR03] Singh D, Lee-Cruz L, Kim WS, Kerfahi D, Chun JH, Adams JM (2014) Strong elevational trends in soil bacterial community composition on Mt. Halla, South Korea. Soil Biol Biochem 68:140–149

[CR27] Singh D, Takahashi K, Kim M, Chun J, Adams JM (2012). A hump-backed trend in bacterial diversity with elevation on Mount Fuji, Japan. Microb Ecol.

[CR28] Wang Q, Garrity GM, Tiedje JM, Cole JR (2007). Naïve Bayesian Classifier for rapid assignment of rRNA sequences into the new bacterial taxonomy. Appl Environ Microbiol.

[CR29] Ward NL, Challacombe JF, Janssen PH, Henrissat B, Coutinho PM, Wu M, Xie G, Haft DH, Sait M, Badger J, Barabote RD, Bradley B, Brettin TS, Brinkac LM, Bruce D, Creasy T, Daugherty SC, Davidsen TM, DeBoy RT, Detter JC, Dodson RJ, Durkin AS, Ganapathy A, Gwinn-Giglio M, Han CS, Khouri H, Kiss H, Kothari SP, Madupu R, Nelson KE, Nelson WC, Paulsen I, Penn K, Ren Q, Rosovitz MJ, Selengut JD, Shrivastava S, Sullivan SA, Tapia R, Thompson LS, Watkins KL, Yang Q, Yu C, Zafar N, Zhou L, Kuske CR (2009). Three genomes from the phylum *Acidobacteria* provide insight into the lifestyles of these microorganisms in soils. Appl Environ Microbiol.

[CR30] Yarwood SA, Myrold DD, Högberg MN (2009). Termination of belowground C allocation by trees alters soil fungal and bacterial communities in a boreal forest. FEMS Microbiol Ecol.

[CR001] Yergeau E, Bezemer TM, Hedlund K, Mortimer SR, Kowalchuk GA, Van Der Putten WH (2010). Influences of space, soil, nematodes and plants on microbial community composition of chalk grassland soils. Environ Microbiol.

[CR31] Zhang L, Xu Z (2008). Assessing bacterial diversity in soil. J Soils Sediments.

[CR32] Zhang LM, Wang M, Prosser JI, Zheng YM, He JZ (2009). Altitude ammonia-oxidizing bacteria and archaea in soils of Mount Everest. FEMS Microbiol Ecol.

